# A Biopersistence Study following Exposure to Chrysotile Asbestos Alone or in Combination with Fine Particles

**DOI:** 10.1080/08958370802259053

**Published:** 2008-09-11

**Authors:** D. M. Bernstein, K. Donaldson, U. Decker, S. Gaering, P. Kunzendorf, J. Chevalier, S. E. Holm

**Affiliations:** 1 Consultant in Toxicology, Geneva, Switzerland; 2 University of Edinburgh, ELEGI Colt Laboratory, Edinburgh, Scotland; 3 RCC Ltd, Toxicology, Füllinsdorf, Switzerland; 4 GSA Gesellschaft für Schadstoffanalytik mbH, Ratingen, Germany; 5 Experimental Pathology Services AG, Muttenz/Basel, Switzerland; 6 Georgia-Pacific, Atlanta, Georgia, USA

## Abstract

In designing a study to evaluate the inhalation biopersistence of a chrysotile asbestos that was used as a component of a joint-compound, a feasibility study was initiated to evaluate the short-term biopersistence of the chrysotile alone and of the chrysotile in combination witht the sanded reformulated joint-compound.

Two groups of Wistar rats were exposed to either 7RF3 chrysotile (Group 2) or to 7RF3 chrysotile combined with aerosolized sanded joint-compound (Group 3). In addition, a control group was exposed to flltered-air.

The chrysotile used in the Ready Mix joint compound is rapidly removed from the lung. The chrysotile alone exposure group had a clearance half-time of fibers L > 20 μm of 2.2 days; in the chrysotile plus sanded exposure group the clearance half-time of fibers L > 20 μm was 2.8 days. However, across all size ranges there was approximately an order of magnitude decrease in the mean number of fibers remaining in the lungs of Group 3 as compared to Group 2 despite similiar aerosol exposures.

Histopathological examination showed that the chrysotile exposed lungs had the same appearance as the flltered-air controls.

This study uniquely illustrates that additional concurrent exposure to an aerosol of the sanded joint-compound, with large numbers of fine-particles depositing in the lungs, accelerates the recruitment of macrophages, resulting in a tenfold decrease in the number of fibers remaining in the lung. The increased number of macrophages in the chrysotile/sanded joint exposure group was confirmed histologically, with this being the only exposure-related histological finding reported.

## Introduction

The inhalation biopersistence study was originally developed in order to quantify the rate at which fibers clear from the lung (Bernstein et al., 1994; Musselman et al., 1994). Since its inception, numerous synthetic and and natural mineral fibers have been evaluated using this protocol design (Bernstein & Reigo, 1999). For synthetic mineral fibers, the biopersistence of the fibers longer than 20 μm was found to be highly correlated with pathological response in chronic inhalation studies, and with tumorigenic potential in chronic intraperitoneal injection studies. More recently, commercial chrysotile, evaluated under the same protocol, was found to clear rapidly from the lungs with clearance half-times of fibers longer than 20 μm ranging from 0.3 to 11.4 days, depending on chrysotile fiber type. However, these studies always involved the exposure of the pure bulk product without the addition of any secondary aerosol to the test atmosphere.

We set out to evaluate the biopersistence of the commercial chrysotile asbestos that was used through the mid-1970s in a joint compound intended for sealing the interface between adjacent wall boards. An inhalation biopersistence study was designed to include not only the chrysotile component of the joint compound alone but also the sanded particulate component, as real-life exposure to abraded wall board would result in concomitant exposure to both chrysotile fibers and joint compound particles. The inclusion of the sanded particulate fraction was considered important in order to determine whether any of the particulate components of the joint compound influenced the biopersistence or pathological response to the chrysotile.

The protocol design, developed for the fiber inhalation biopersistence study, recommended that the fiber exposure aerosol should be prepared to be rat-respirable and should have at least 100 fibers per cubic centimeter longer than 20 μm in length (Bernstein & Riego-Sintes, 1999). In addition, the geometric mean diameter of those fibers longer than 20 μm should be as close to 0.8 μm as possible (for fibers with a density ρ ≈ 2.4) in order to assure that they were rat-respirable. However, the joint compound contained less than 5% chrysotile and the chrysotile grade consisted of very short fibers. In addition, the particles often agglomerated into larger particles which were not ratrespirable (aerodynamic diameter less than ≈3 μm). As a result, it would not be feasible to generate a test atmosphere for an inhalation biopersistence study that met the protocol criteria of having at least 100 fibers with lengths greater than 20 μm per cubic centimeter of air and expose the rats to a representative sanded joint compound particulate component as well.

In order to address these issues and conduct an inhalation biopersistence study on the sanded joint compound, the chrysotile and the powder components of the formulation were aerosolized separately and then mixed in the airstream prior to delivery to the rats as discussed below. As this was the first time that such a mixed exposure was performed following the fiber inhalation biopersistence protocol guidelines (Bernstein & Riego, 1999), a feasibility study was initiated in order to validate the aerosol generation and exposure systems for each component.

## Methods

### Chrysotile and Powder Sample Characteristics

#### Chrysotile Sample

The original formulations for the Ready Mix that was used to produce the sanded material specified chrysotile asbestos grade 7RF9, which was obtained historically from the Philip Carey mine in Canada. Because this mine is no longer in operation, an alternative type of asbestos was used in the reformulation. The asbestos used was selected following extensive characterization of the chrysotile in a sample of historical joint compound and then comparing this to three commercially available Grade 7 chrysotile asbestos samples: Johns Manville (JM) 7RF3, JM 7R-05, and Brazilian CB7-RP (Brorby et al., 2008). The authors reported that replicates of JM 7RF3 were considered to be most similar to the length and diameter distribution of the chrysotile in the historical Carey 7RF9.

#### Sanded M971/974 Powder

The Ready Mix joint compound was reformulated as described by Brorby et al. (2008). The ingredients of the reformulated compound are shown in Table 1, reproduced from Brorby et al. (2008).

**TABLE 1:** Ready Mix ingredients

Ingredient	Function	Mass used in reformulation (g)	Percent by weight	Supplier
Filler				
Limestone^b^	Filler	5255	81	Science Stuff
Mica^c^	Anti-cracking agent	845	13	Zemex Industrial Minerals
Natrosol	Water retention agent	42.25	0.65	Kings Mountain Mining, LLC, Aqualon
Gelvatol 20-30 BP (Polyvinyl alcohol)	Adhesive	52	0.8	Science Laboratories, Alfa Aesar
Troysan CMP-10-Sep^a^	Fungicide	—	—	
Dowicil	Substitute for Troysan CMP-10-Sep	9.75	0.15	Dow Chemical Co.
Nalco 71-D5^a^	Anti-foaming agent	—	—	—
Chrysotile 7RF9^a^	Bulk	—	—	—
Chrysotile 7RF3	Substitute for 7RF9	292.5	4.5	Johns Manville
Ready Mix				
Filler	Body	5485	57	—
Elvacet 81-900 (polyvinyl acetate emulsion)	Adhesive	—	—	—
Playamul 104	Substitute for Elvacet 81-900	482	5	Forbo
Benzoflex 50	Plasticizer	31.2	0.3	Velsicol
Nalco 71-D5^a^	Antifoaming agent	6	0.06	Nalco
Water	Solvent	3602.5	37.5	Municipal Supply

a Ingredient substituted or eliminated—see Brorby et al. (2008).

b Limestone: 100% calcium carbonate natural chalk (CAS number 471-34-1), principal component of limestone is the mineral calcite: CaCO_3_.

c Chemical composition of mica: muscovite, K_2_Al_4_Si_6_Al_2_O_20_(OH,F)_4_.

The reformulated compound was applied according to the instructions of the original material to pieces of drywall, the ends of which were sealed with tape. A notched trowel was used for application. The material was allowed to dry for at least 48 h and then sanded. Individual boards were sanded for ≈ 20–30 min. Four different boards were needed to obtain sufficient mass of material for the studies. Aluminum oxide medium 120-grit sand paper was used to generate the sample. The sanded material was collected in a large Ziploc bag and the bag was sent to the Research and Consulting Company Ltd. (RCC), Füllinsdorf, Switzerland, where the biopersistence feasibility study was performed.

#### Chrysotile Mineralogical Grade

The fiber used in this evaluation has the mineralogical grade of 7R.

The determination of fiber grade is based upon the Canadian chrysotile asbestos classification. This classification was established by the Committee on Uniform Classification and Grading of Asbestos Mines Products. This committee was formed in 1931 at the instigation of the Minister of Mines of the Province of Quebec (Cossette & Delvaux, 1979).

Milled asbestos consist of all grades produced by the mechanical treatment of asbestos ore. The milled asbestos grades are classified into groups 3 to 7 using a Quebec Standard Testing Machine, which actively sieves the fiber through different mesh screens using a rotating sieve shaker. This machine consists of a nest of four boxes (or trays) superimposed one above the other. The bottoms of the first three boxes (numbers 1, 2, and 3) are made of bronze screening, each of successively smaller screen size.

The test specimen is spread on the uppermost tray (number 1), which is then covered, and tightly c1amped. The machine is started, and is kept operating by an automatic device until 600 revolutions have been completed. At the end of the sifting action, the fiber retained on each tray is weighed. The longest fiber naturally stays on the screen with the largest opening, whereas shorter fiber, according to its length, remains on screen (or tray) 2 or 3, or drops into the pan (lowest tray). The more fiber retained on the first screen, and the less fiber falling into the pan, the higher the grade. The mesh size of the smallest screen (tray 3) is 10, which corresponds to a nominal sieve opening of 1.70 mm.

Grades 7D to 7T (which includes 7R) are generally termed “shorts” because they contain primarily shorter fibers and have been primarily used in the role of a fibrous mineral filler in a variety of manufactured products including the joint compound. This shorter material is often comprised of bundles of short fibers. A cyclone separator was included in the aerosol generation stream in order to remove some of the thicker bundles, which would not be respirable in the rat.

#### Fiber Composition

The chemical composition of chrysotile is shown in Table 2. Chrysotile is composed of a silicate sheet of composition (Si_2_O_5_)_n_^−2n^, in which three of the O atoms in each tetrahedron are shared with adjacent tetrahedra and a nonsilicate sheet of composition [Mg_3_O_2_(OH)_4_]^n+2n^. In chrysotile the distances between apical oxygens in a regular (idealized) silicate layer are shorter (0.305 nm) than the O–O distances in the ideal Mg-containing layer (0.342 nm), which may account for the curling of the layers, which results in the rolling up like a carpet to form concentric hollow cylinders (Skinner et al., 1988). The Mg molecule is on the outside of the curl and is thus exposed to the surrounding environment of the lung fluid. Hume and Rimstidt (1992) have described the dissolution of chrysotile (Thetford Mine) as occurring in two steps. First the magnesium hydroxide layer, which is closest to the fiber surface (Smith, 1974), is removed by rapid leaching; then the silica layer dissolves at a slower rate. The acid pH of the macrophage vacuole may accelerate this process and/or destabilize the chrysotile structure, allowing for either separation into the smaller base fragments or breakage of the longer fibers.

**TABLE 2:** Typical chrysotile chemical composition (percent)

Compound	Chrysotile^a^
SiO_2_	39.77
Al_2_O_3_	0.66
Fe_2_O_3_	2.02
MnO	0.07
MgO	40.62
CaO	0.32
Na_2_O	0.01
H_2_O^+^	12.69
H2	1.54
CO_2_	0.78
Total	98.4

a Campbell et al. (1980).

### Experimental Design

The experimental design of the in-life and biopersistence analysis has been presented in detail previously (Bernstein et al., 1994; Bernstein & Riego, 1999) and is summarized next. In particular, details of the counting and sizing procedures are reiterated as these are considered essential to the successful interpretation of these studies.

#### Animal Exposure

Three groups of laboratory rats (groups 1, 2 and 3) were be exposed for 6 h per day for 5 days to:

Group 1: Filtered air alone (negative control group).

Group 2: A fixed exposure level of well-characterized fibers of chrysotile 7RF3.

Group 3: A fixed exposure level of well-characterized fibers of chrysotile 7RF3 mixed with sanded M971/974 powder.

Groups of 14 weanling (8–10 wk old) male rats were exposed by flow-past nose-only exposure for 6 h/day for a period of 5 consecutive days. In groups 2 and 3, a chrysotile concentration higher than that required by the EC Biopersistence Protocol (Bernstein & Riego, 1999) of 100 fibers with length *L* > 20 μm/cm^3^ was used in order to assure there was sufficient long-fiber exposure. To be comparable with current and previous fiber inhalation studies, Wistar rats (HanBrl:WIST, specific pathogen free) were used and were obtained from RCC Ltd, Biotechnology and Animal Breeding Division, CH-4414 Füllinsdorf, Switzerland.

A negative control group (group 1) was exposed in a similar fashion to filtered air. These animals were included as histopathological controls for comparison to groups 2 and 3.

#### Exposure System

The fiber aerosol generation system was designed to loft the bulk fibers without breaking, grinding, or contaminating the fibers (Bernstein et al., 1994). The animals were exposed by the flow-past nose/snout-only inhalation exposure system. This system was derived from Cannon et al. (1983) and is different from conventional nose-only exposure systems in that fresh fiber aerosol is supplied to each animal individually and exhaled air is immediately exhausted.

The bulk chrysotile 7RF3 fiber was observed to have numerous bundles of fibers. While this is characteristic of this grade of fiber, it was necessary to open up the bundles to generate an aerosol that would be respirable by the rats. This was accomplished by passing the bulk fiber through a Cyclotec mixer (Trecator SA) for 2 periods of 5 min each. This device is designed to minimize degradation of the sample. The sample is rolled against the inner circumference of a durable surface and passed out through a screen.

**FIG. 1:**
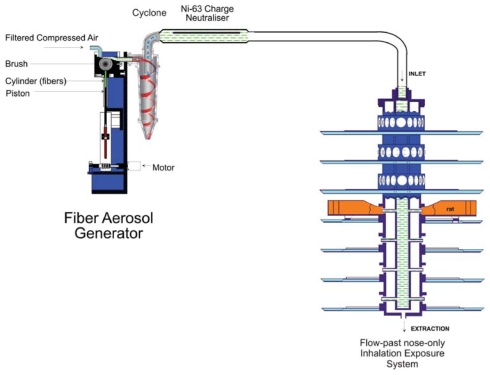
Fiber aerosol generation and exposure system used for group 2, chrysotile. The fiber aerosol is generated using a rotating-brush-feed aerosol generator. Immediately following the generator, the airstream is passed though a cyclone to remove larger particles and fibers that would not be rat-respirable. The aerosol then passes through an Ni-63 charge neurtralizer and is then fed into the flow-past nose-only aerosol exposure system.

The chrysotile aerosol was generated using a rotating brush aerosol generator as shown in Figure 1. The aerosol generator was followed by a 500-ml Pyrex glass cyclone to assist further in the elimination of fiber bundles from the aerosol and then by a ^63^Ni charge neutralizer to reduce electrostatic charge on fibers to Boltzmann equilibrium.

**FIG. 2:**
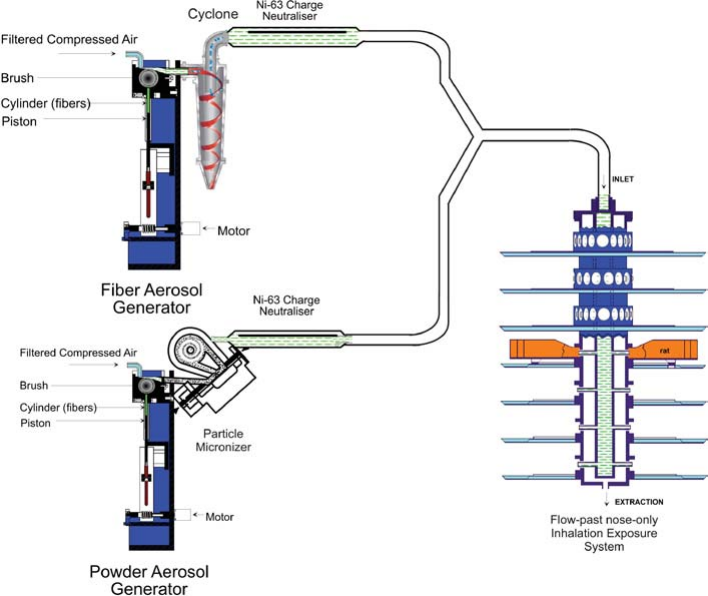
Dual aerosol generators for fiber and powder generation and exposure system used for group 3, chrysotile and sanded material. The fiber generation system is identical to that described for Figure 1. The powder aerosol (sanded material) is generated using a rotating-brush-feed aerosol generator. Immediately following the generator, the airstream is fed to an in-line micronizing system that assures that the aerosol is rat-respirable. The aerosol then passes through an Ni-63 charge neurtralizer. The two aerosol streams are mixed and the combined fiber and sanded material aerosol is then fed into the flow-past nose-only aerosol exposure system.

For group 3 (chrysotile fiber 7RF3 mixed with sanded M971/974 powder), a fiber aerosol was generated from chrysotile fiber 7RF3 and separately a dust aerosol from sanded M971/974 using individual rotating-brush aerosol generators (Figure 2). The aerosol generator used for generation of the fiber aerosol was followed by a 500-ml Pyrex glass cyclone to assist in the elimination of fiber bundles from the aerosol; the aerosol generator used for generation of the dust aerosol was followed by a micronizing jet mill to reduce the particle size. Following each generator, in-line 63Ni charge neutralizers reduced the electrostatic charge from fibers and particulate material in the generated aerosols.

Following the charge neutralizers, the fiber and powder aerosols were mixed through a Y-piece connection and then delivered directly into the nose-only flow-past exposure chamber.

Control animals (group 1 air control) were treated with filtered air passed through a separate brush-feed generator.

#### Exposure System Monitoring

The aerosol mass was sampled for approximately 5 h during each exposure. Aerosol samples were collected on the appropriate filters in the vicinity of the animal's snout. Likewise, the temperature, relative humidity, and oxygen concentration were measured on atmosphere/aerosol samples collected directly from the delivery tube in the breathing zone of the animals. In addition, in group 3 (chrysotile fiber 7RF3 mixed with sanded M971/974 powder), aerosol samples were also taken from a sampling outlet following the micronising jet mill, in order to monitor the proportion of particulate material derived from the dust aerosol generation from sanded M971/974 powder in the test aerosol administered to the animals of this group.

#### Gravimetric Determination of Aerosol Concentrations

Gravimetric determinations of aerosol concentration were performed at least once daily for each of groups 2 and 3 using a Millipore durapore filter (type HVLP, polyvinylidene difluoride membrane, pore size 0.45 μm), loaded in a 47-mm in-line stainless-steel filter sampling device.

#### Fiber Number and Size Distribution of Aerosol Concentrations

For bivariate analysis of fiber size distribution and counting, aerosol samples were collected in the vicinity of the animal's snout onto Nuclepore filters (PC membrane, diameter 47 mm, pore size 0.2 μm; SN 111.106, Nuclepore Ltd.). On each occasion two samples (one main sample and one reserve) were taken on Nuclepore filters for bivariate analysis of fiber size distribution and counting. These samples were taken for approximately 5 h during each exposure period in parallel with the sampling scheduled for gravimetric determination of aerosol concentrations. The bivariate analysis of fiber size distribution and counting (expressed in fiber number per cubic centimetre of aerosol) was performed by GSA, Gesellschaft für Schadstoffanalytik mbH (Germany), using analytical scanning transmission electron microscopy (ASTEM) with energy-dispersive x-ray analysis (EDS).

#### Lung Digestion for Fiber/Particle Analysis

The lungs from 3 rats per group per time point were frozen at necropsy at minus 20°C. The frozen lungs were subsequently dehydrated by freeze drying (Edwards EF4 Modulyo freeze dryer) and dried to constant weight to determine the dry weight of the tissue. The dry tissue was plasma-ashed in a Plasma systems 200 (Technics Plasma GmbH) for at least 16 h. Upon removal from the ashing unit, the ash from each lung was weighed and suspended in 10 ml methanol using a low-intensity ultrasonic bath. The suspension was then transferred into a glass bottle with the combustion boat rinse and the volume made up to 20 ml. An aliquot was then removed and filtered onto a gold-coated and uncoated polycarbonate filter (pore size of 0.2 μm).

#### Counting Rules for the Evaluation of Air and Lung Samples by Transmission Electron Microscopy

All fibers visible at a magnification of 10,000 × were taken in consideration. All objects seen at this magnification were sized with no lower or upper limit imposed on either length or diameter. The bivariate length and diameter was recorded individually for each object measured. Fibers were defined as any object that had an aspect ratio of at least 3:1. The diameter was determined at the greatest width of the object. All other objects were considered as nonfibrous particles.

The stopping rules for counting each sample were defined as follows.

#### Fibrous Particles

For the fiber measurement a sizeselective analysis was used. Three length categories were defined:

- Fibers with a length < 5 μm.
- Fibers with a length between 5 and 20 μm.
- Fibers with a length > 20 μm.

Fields of view were examined for each length category until the defined minimum of fibers for each length categorywas recorded or a maximum of 1 mm^2^ of the filter surface was examined in case the fiber minimum for the length category was not reached.

The minimum of fibers to examine for the three length categories was:

- For fibers with a length <5 μm, 100 fibers (200 fiber ends).
- For fibers with a length between 5 and 20 μm, 200 fibers (400 fiber ends).
- For fibers with a length >20 μm, 100 fibers (200 fiber ends).

#### Nonfibrous Particles

Fields of view were examined until the aforementioned stopping criteria for fibers was reached or a total of 100 particles was recorded.

For samples of the control group only an area of 0.5 mm^2^ of the filter was evaluated.

#### Particle Size of Dust Aerosol

Particle size distribution of the dust aerosol generated from sanded M971/974 powder during the exposures of group 3 (chrysotile fiber 7RF3 mixed with sanded M971/974 powder) was measured gravimetrically during the aerosol generation setup prior to the 5-day treatment period using a 7 stage cascade Mercer impactor (model 02-130, In-Tox Products, Inc., Albuquerque, NM). Mass median aerodynamic diameters (MMAD) and geometric standard deviations (GSD) were calculated on the basis of the results from the Mercer impactor, using Microsoft Excel software. The targetwas to produce a rat-respirable aerosol in the range 1 to 4 μm MMAD.

#### Clinical Examination and Body Weights

The animals were observed for mortality/morbidity at least once daily during the acclimatization period, twice daily before and after exposure and during the treatment period, and once daily during the 3-day postexposure observation period.

Clinical signs were recorded on the first day of the acclimatization period, twice daily during the treatment period (once preexposure and once postexposure outside the restraint tubes), and once daily during the postexposure observation period.

During exposure, only grossly abnormal signs were visible, as the animals were in restraint tubes. Observations were detailed and carefully recorded using explicitly defined scales as appropriate. Observations included, but were not be limited to, changes in behavior, somatomotor activity and body position, respiratory and circulatory effects, autonomic effects such as salivation, central nervous system effects, e.g., tremors or convulsions, reactivity to handling or sensory stimuli, altered strength, and alteration of the skin, fur, nose, eyes and mucous membranes.

Each animal was weighed twice during the acclimatization period (days 1 and 5), twice during the treatment period (prior to tube restraint on treatment days 1 and 5), and again on day 5 of the post observation period.

#### Histopathology

At 0 days (immediately after the cessation of exposure) and at 3 days postexposure, separate subgroups of animals (3 animals per group) were taken for histopathological examination of the respiratory tract. Following resection, the lungs and mediastinal lymph nodes were processed, embedded in paraffin, cut at a nominal thickness of 2–4 μm, and stained with haematoxylin and eosin for histopathological examination.

#### Fiber Clearance

At 0 days (immediately after the cessation of exposure) and at 3 days postexposure, the lungs from subgroups of animals were weighed and then digested by low-temperature plasma ashing and subsequently analyzed by transmission electron microscopy (at the GSA Corp.) for total chrysotile fiber number in the lungs and chrysotile fiber size (bivariate diameter) distribution in the lungs. The mediastinal tissue containing the mediastinal lymph nodes was resected and discarded.

The dissection of the respiratory tract was performed under a dissecting microscope. All procedures were designed so as to avoid contamination of the dissected organs by fibers from the fur or fibers that might be deposited on dissecting instruments.

The lung lobes were weighed together and inserted into an appropriately labeled plastic bag and deep frozen without fixation. They were subsequently transferred from dry ice to a refrigerator at −20 ± 5°C until digested. The frozen lung lobes were dried in an Edwards EF Modulyo freeze dryer. The lung dry weight was calculated by deducting the weight of the empty vessel from the weight of the vessel containing the thawed lung after drying. The dry tissue from all lungs was plasma ashed in a Plasma Systems 200 multiple-chamber unit from Technics Plasma GmbH. For each lung, the ash weight was calculated by weighing the respective vessel before and after the ashing procedure.

Upon removal from the ashing unit, the lung ash was retained in the same vials without transfer. These vials were then sealed and express mailed under ambient conditions to GSA (Gesellschaft für Schadstoffanalytik mbH) for bivariate analysis of fiber size distribution and counting using ASTEM-EDS.

## Results

### Validation of the Lung Digestion and Counting Procedures

Validation of lung digestion and counting procedures is essential to the legitimacy of this type of study, although it was often absent from early studies. Such validation provides confidence that there is no significant alteration of the fiber length distribution during fiber recovery.

The only method suitable for validation of chrysotile fiber recovery would be a parallel analysis using a noninvasive measurement technique such as confocal microscopy and comparison of these results with those obtained following transmission electron microscopy examination of the digested lungs. This type of comparative analysis of the fiber number and size distribution is planned for the main study. Previous studies using the same procedures used here have confirmed that the fiber recovery procedures do not significantly alter the fiber size distribution or number of the chrysotile fibers (Bernstein et al., 2004, 2005a, 2005b).

### Inhalation Exposure

The inhalation biopersistence protocol that has been recommended by the ILSI working group (ILSI, 2005) and adapted by the European Commission as part of its synthetic fiber directive (European Commission, 1997) specifies that the atmosphere to which the animals are exposed should have at least 100 fibers/cm^3^ longer than 20 μm. As examination of the fiber sample indicated that longer fibers were sometimes present as strands of multiple fibers, in this study, the number of fibers per cubic centimeter longer than 20 μm was purposely increased in order to maximize the number of longer fibers in the exposure atmosphere.

The exposure concentration used in the studywas determined through an iterative process, which involved the exposure of groups of three rats for a single 6-h period followed by sacrifice after cessation of exposure. The lungs were removed, freezedried, digested by low-temperature ashing, and subsequently analyzed by transmission electron microscopy in order to determine the number of fibers longer than 20 μm that deposited in the lungs. With this procedure, an exposure concentration was chosen that assured that a sufficient number of rat-respirable longer fibers were present in the aerosol so that their clearance could subsequently be measured from the lung.

The number, concentration, and size distribution of the chrysotile (group 2), and chrysotile plus sanded powder exposure group (group 3) are shown in Table 3. The mean number of WHO fibers (defined as fibers >5 μm long, <3 μm wide, and with length:width ratios >3:1; WHO, 1997) in the group 2 and group 3 exposure atmospheres was 4496 fibers/cm^3^ and 4944 fibers/cm^3^, respectively. This exposure was more than 40,000 times the OSHA occupational exposure limit for chrysotile of 0.1 fibers/cm^3^. The mean number of fibers longer than 20 μm was 1055 fibers/cm^3^ and 912 fibers/cm^3^ in group 2 and group 3, respectively.

**TABLE 3:** The number, concentration, and size distribution of the chrysotile and chrysotile and powder exposure atmospheres

Exposure group	Gravimetric concentration (mg/m^3^), mean ± standard deviation	Mean number of fibers evaluated	Mean number of total fibers per cm^3^	Mean number WHO fibers per cm^3^	Mean percent WHO fibers of total fibers	Mean number of fibers with L > 20 μm per cm^3^	Mean percent fibers L > 20 μm of total fibers	Diameter range (μm)	Length range μm)	GMD^1^ (μm)/GSD^2^	GML^3^ (μm)/GSD^2^	Mean Diameter (μm) ± Standard Deviation	Mean Length (μm) ± Standard Deviation
Group 2: Chrysotile fiber concentration:	3.5 ± 0.2	410	12,423	4496	35	1055	8	0.01-1.2	1.0-115	0.04/2.2	4.4/2.7	0.05 ± 0.06	7.4 ± 10.4
Group 3: Chrysotile fiber concentration:	3.2 ± 0.3	406	18,515	4944	26	912	5	0.01-0.8	1.0-120	0.03/2.1	3.8/2.3	0.05	5.8 ± 8
Powder concentration	1.3 ± 0.2	—	—	—	—	—	—	—	—	—	—	—	—

1 GMD = geometric mean diameter.

2 GSD = geometric standard deviation.

3 GML = geometric mean length.

#### Particle Number Concentration

Table 4 presents a summary of the mean number of particles (i.e. objects with an aspect ratio <3:1) in the exposure aerosol per cubic centimeter air as determined by TEM according to the counting criteria outlined in the Methods section. In group 3, due to the addition of the powder, the mean number of particles/cm3 was 110 particles/cm^3^ air, nearly 3 times that of group 2.

**TABLE 4:** Mean number of particles^a^ in the aerosol as evaluated by TEM

Parameter	Group 2, chrysotile	Group 3, chrysotile and powder^b^
Mean number of particles per cm^3^ air	38	(110) 1500^b^
≤1 μm particles per cm^3^ air	1	15
>1 μm to ≤3 μm particles per cm^3^ air	32	86
>3 μm particles per cm^3^ air	4	9

a Particle was considered as an object with an aspect ratio <3:1.

b As presented later, SEM/EDAX analysis of the particles in the aerosol and lung of groups 2 and 3 has shown that the sample preparation process has dissolved the limestone particles that make up over 80% of the filler material in the joint compound (Table 1). Calculation of the number of particles that would be present based upon the mass concentration and the size distribution indicates that there were approximately 1500 partciles/cm^3^ (calculated using the Aerosol Calculation Software by Paul Baron).

Particle size analysis of the sanded M971/974 powder using a Mercer cascade impactor sampled directly following the aerosol micronizer indicated that the MMAD was 1.18 μm (GSD: 2.75). The analysis showed that84%of the particles were rat-respirable (≤3 μm).

#### Chemical Identification of the Particles (EDAX Analysis)

**FIG 3:**
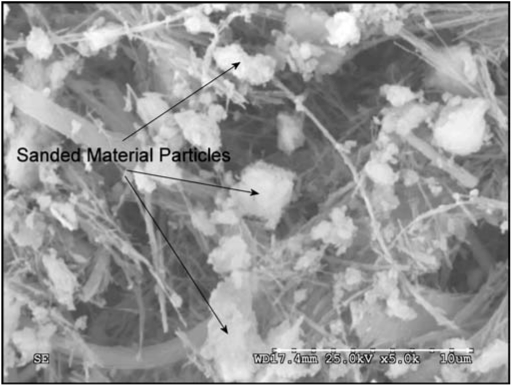
SEM photomicrograph of one of the reserve aerosol sampling filters from exposure group 3 (chrysotile and sanded material). As can be seen, the particle/fiber loading of the filter was too dense to allow determination of the concentration; however, numerous particles of the sanded material are visible as well as the chrysotile fibers. The EDAX analysis of the particles/fibers in the center of the photomicrograph is shown in Figure 4.

**FIG 4:**
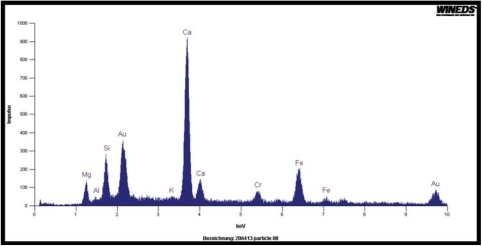
EDAX analysis of the particles/fibers in the center of the photomicrograph in Figure 3. The predominant peak is Ca from the calcium carbonate (limestone) in the sanded material. The Mg and Si are from the chrysotile. The smaller amounts of Fe, Cr, and K are also from the sanded material. The small amount of Al is probably from the sandpaper. The Au is used to coat the filter to permit SEM/EDAX analysis. It should be noted that while the Si, Mg, and Ca appear together in this spectra taken from the aerosol filter, in the actual aerosol, the sanded material (Ca) and the chrysotile (Si, Mg) are each generated independently and then mixed in the airstream (Figure 2). Each aerosol stream was charge neutralized to Boltzmann equilibrium and from the time of mixing the combined aerosol is delivered to the rat's nose in approximately 3-4 s.

In order to determine the elemental composition of the particles in the aerosol and those recovered from the lung, one aerosol filter and one lung sample at day 0 from groups 2 and 3 were analyzed by SEM/EDAX. In total, 300 particles were analyzed from each sample. SEM/EDAX analysis of the particles in the aerosol and lung of groups 2 and 3 has shown that the sample preparation process has dissolved the limestone particles that make up over 80% of the filler material in the joint compound (Table 1). This was due to the use of methanol, which was initially chosen because the fibers are not soluble in methanol. However, we subsequently found that the presence of methanol increases the solubility of carbon dioxide, which has the effect of increasing the solubility of the calcium carbonate particles. The presence of the calcium carbonate particles was confirmed by performing SEM/EDAX analysis directly on one of the reserve aerosol sampling filters from exposure group 3 (chrysotile and sanded material). The particle/.ber loading of the filter was too dense to allow determination of the concentration; however, as shown in the SEM photomicrograph (Figure 3), there are numerous particles of the sanded material as well as chrysotile fibers. The composition of the particles was confirmed by EDAX as shown in Figure 4.

In order to estimate the number of calcium carbonate particles that were present in the aerosol, the numbers of particles were determined based upon the mass concentration and the size distribution. Using the Aerosol Calculation Software by Paul Baron (www.bgiusa.com) it was calculated that there were approximately 1500 particles/cm^3^ present in the aerosol.

#### Bivariate Length and Diameter Distribution

**FIG 5:**
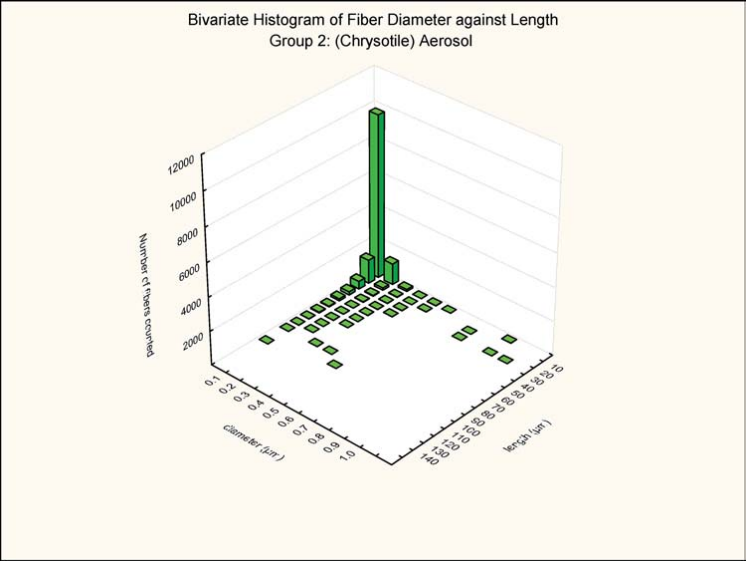
Group 2, chrysotile aerosol, bivariate length and diameter distribution of each fiber and particle measured during the 5-day exposure.

**FIG 6:**
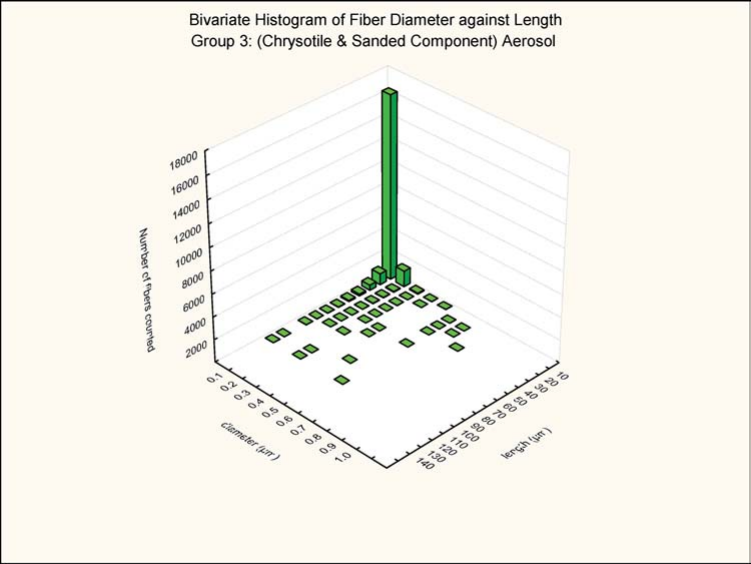
Group 3, chrysotile and sanded component aerosol, bivariate length and diameter distribution of each fiber and particle measured during the 5-day exposure.

The bivariate length and diameter distribution of each fiber measured according to the counting rules already given was recorded. The aerosol generation technique was designed to maximize the number of long respirable fibers. As illustrated in Figures 5 and 6 for the group 2 chrysotile and group 3 mixed chrysotile and powder exposure, respectively, 98% of the chrysotile fibers in the exposure atmosphere were less than 0.2 μm in diameter and thus potentially respirable in the rat. Of the longer fibers (L > 20 μm), 98% of the long chrysotile fibers were less than 0.2 μm in diameter in both groups. There were no statistically significant differences in the number and size distributions of the chrysotile components of the two exposure atmospheres.

### Lung Fiber Burden

The number, concentration, and size distribution of the chrysotile fibers in lungs of the rats from group 2 chrysotile exposure and group 3 chrysotile and sanded component immediately after the termination of the 5-day exposure (day 0) and at 3 days following cessation of exposure (day 3) are presented in Tables 5 and 6, respectively. The data for each animal individually and group means are shown for each parameter.

As shown in Table 5, immediately following cessation of exposure (0 days), there was a mean of 27.2 million WHO fibers remaining in the lungs of rats in group 2, which received only chrysotile. Rats from group 3, which received both chrysotile and the sanded powder, had only 2.3 million WHO fibers at the same time point (0 days). Similarly, there were a mean of 0.44 million fibers longer than 20 μm in length from rats of group 2, and a mean of 0.037 million fibers longer than 20 μm in length from rats of group 3.

**TABLE 5:** Number, concentration, and size distribution of the chrysotile fibers in lungs of the rats from group 2, chrysotile exposure, and group 3, chrysotile and sanded component, immediately after the termination of the 5-day exposure (day 0)

	Group 2, chrysotile 0 Days after cessation of exposure				Group 3, chrysotile and sanded 0 Days after cessation of exposure			
		
Lung samples	Animal 9	Animal 10	Animal 11	Mean	Animal 23	Animal 24	Animal 25	Mean
Fibers								
Number of fibers evaluated	328.5	318.5	325.0	—	307.5	308.5	215	—
Number of total fibers (millions/lung lobes)	231.4	151.9	155.4	179.6	15.5	11.2	8.1	11.6
Number of WHO^b^ fibers (millions/lung lobes)	36.2	24.3	21.0	27.2	3.0	2.6	1.3	2.3
Number of WHO^b^ fibers of total fibers (%)	15.7%	16.0%	13.5%	15.1%	19.5%	23.2%	15.8%	19.8%
Number of fibers *L* > 20 μm (millions/lung lobes)	0.53	0.38	0.41	0.44	0.06	0.03	0.02	0.04
Fibers *L* > 20 μm of total fibers (%)	0.2%	0.2%	0.3%	0.2%	0.4%	0.3%	0.3%	0.3%
Number of fibers *L* 5–20 μm (millions/lung lobes)	35.7	23.9	20.6	26.7	3.0	2.6	1.3	2.3
Fibers *L* 5–20 μm of total fibers (%)	15.4%	15.7%	13.2%	14.9%	19.1%	22.9%	15.5%	19.5%
Number of fibers *L* ≤5 μm (millions/lung lobes)	195.2	127.7	134.4	152.4	12.5	8.6	6.8	9.3
Fibers L ≤5 μm of total fibers (%)	84.3%	84.0%	86.5%	84.9%	80.5%	76.8%	84.2%	80.2%
Diameter range (μm)	0.01-0.6	0.01-1.5	0.01-1.1	0.01-1.5	0.01-0.7	0.01-0.6	0.01-0.5	0.01-0.7
Length range (μm)	1.0-63.0	1.0-65.0	1.0-60.0	1.0-65.0	1.0-30.0	1.0-35.0	1.0-24.0	1.0-35.0
Mean diameter (μm)	0.07	0.07	0.07	0.07	0.10	0.09	0.10	0.10
SD	0.06	0.07	0.08	0.07	0.10	0.08	0.10	0.09
Mean length (μm)	3.76	3.97	3.68	3.79	3.94	3.90	3.44	3.81
SD	2.69	2.97	2.71	2.78	2.94	2.84	2.57	2.83
GMD (μm)	0.05	0.05	0.05	0.05	0.07	0.06	0.07	0.06
GSD	2.39	2.41	2.35	2.38	2.60	2.48	2.58	2.56
GML (μm)	3.16	3.36	3.12	3.20	3.31	3.25	2.88	3.19
GSD	1.78	1.73	1.71	1.75	1.74	1.78	1.74	1.76
Mode diameter (μm)	0.03	0.03	0.03	0.03	0.03	0.03	0.03	0.03
Mode length (μm)	2.0	2.5	2.0	2.0	2.5	2.0	2.0	2.0
Median diameter (μm)	0.04	0.04	0.04	0.04	0.05	0.05	0.05	0.05
Median length (μm)	3.5	3.5	3.0	3.5	3.0	3.0	2.5	3.0
Aspect ratio mean	98.0	101.8	89.2	96.6	74.0	85.0	65.8	75.6
Total length	870.2	602.8	571.1	681.4	61.0	43.6	27.8	44.1
Particles								
Number of particles evaluated	100.0	100.0	100.0	—	102.0	101.0	104.0	—
Mean number of particles (millions/lung lobes)	3.35	3.47	3.17	3.33	9.39	7.44	6.13	7.65^a^
≤1 μm particles (millions/lung lobes)	0.60	0.80	0.60	0.67	1.66	1.62	1.00	1.43
>1 μm to ≤3 μm particles (millions/lung lobes)	2.64	2.43	2.35	2.48	7.37	5.52	4.77	5.89
>3 μm particles (millions/lung lobes)	0.10	0.24	0.22	0.19	0.37	0.29	0.35	0.34

a The mean particle lung burden in group 3 does not include the large number of CaCO_3_ (limestone) particles that the rats in group 3 were exposed to, which as described earlier could not be counted as they were dissolved by the analytical procedure.

b WHO fibers are defined as having a length greater than 5.0 μm, a diameter less than 3.0μ m, and an aspect ratio equal to or greater than 3:1 (WHO, 1985).

Across all size ranges there was approximately an order of magnitude difference in the mean number of each size category of fibers remaining in the lungs of group 2 as compared to group 3.

These differences between group 2 and group 3 were similar at 3 days postexposure, as shown in Table 6. At that time point, there was a mean of 0.17 million fibers longer than 20 μm in length in rats from group 2, while there were only 0.018 million fibers longer than 20 μm in length in rats from group 3.

**TABLE 6:** Number, concentration and size distribution of the chrysotile fibers in lungs of the rats from group 2, chrysotile exposure, and group 3, chrysotile and sanded component, at 3 days following cessation of exposure (day 3)

	Group 2, chrysotile 3 Days after cessation of exposure				Group 3, chrysotile and sanded 3 Days after cessation of exposure			
		
Lung samples	Animal 16	Animal 17	Animal 18	Mean	Animal 30	Animal 31	Animal 32	Mean
Fibers								
Number of fibers evaluated	308.5	308.0	321.5	—	313	308.5	310.5	—
Number of total fibers (millions/lung lobes)	88.6	88.3	125.4	100.7	13.2	8.4	15.4	12.3
Number of WHO^b^ fibers (millions/lung lobes)	12.7	13.9	26.4	17.6	1.9	1.5	2.3	1.9
Number of WHO^b^ fibers of total fibers (%)	14.3%	15.7%	21.0%	17.5%	14.0%	17.7%	14.9%	15.2%
Number of fibers *L* > 20 μm (millions/lung lobes)	0.11	0.18	0.22	0.17	0.02	0.02	0.02	0.02
Fibers *L* > 20 μm of total fibers (%)	0.1%	0.2%	0.2%	0.2%	0.1%	0.2%	0.1%	0.1%
Number of fibers *L* 5-20 μm (millions/lung lobes)	12.6	13.7	26.2	17.5	1.8	1.5	2.3	1.9
Fibers *L* 5-20 μm of total fibers (%)	14.2%	15.5%	20.9%	17.3%	13.9%	17.5%	14.8%	15.1%
Number of fibers *L* ≤5 μm (millions/lung lobes)	75.9	74.4	99.0	83.1	11.4	6.9	13.1	10.5
Fibers *L* ≤5 μm of total fibers (%)	85.7%	84.3%	79.0%	82.5%	86.0%	82.3%	85.1%	84.8%
Diameter range (μm)	0.01-2.0	0.01-2.0	0.01-0.6	0.01-2.0	0.01-0.5	0.01-0.9	0.01-0.8	0.01-0.9
Length range (μm)	1.0-25.0	1.0-40.0	1.0-45.0	1.0-45.0	1.0-30.0	1.0-50.0	1.0-45.0	1.0-50.0
Mean diameter (μm)	0.07	0.08	0.07	0.07	0.08	0.09	0.08	0.08
SD	0.09	0.10	0.07	0.08	0.09	0.11	0.08	0.09
Mean length (μm)	3.82	3.95	4.57	4.17	3.79	3.76	3.79	3.78
SD	2.35	2.66	2.84	2.67	2.27	2.77	2.47	2.48
GMD (μm)	0.05	0.05	0.04	0.05	0.05	0.06	0.05	0.05
GSD	2.37	2.42	2.35	2.38	2.50	2.47	2.46	2.48
GML (μm)	3.33	3.38	3.99	3.61	3.33	3.17	3.29	3.28
GSD	1.67	1.71	1.65	1.71	1.64	1.75	1.66	1.68
Mode diameter (μm)	0.03	0.03	0.03	0.03	0.03	0.03	0.02	0.03
Mode length (μm)	2.5	2.5	5.0	2.5	2.0	3.5	2.5	2.5
Median diameter (μm)	0.03	0.03	0.03	0.03	0.04	0.05	0.04	0.04
Median length (μm)	3.0	3.5	4.0	3.5	3.5	3.0	3.0	3.0
Aspect ratio mean	98.2	91.6	129.6	109.3	88.5	76.0	88.2	85.6
Total length	338.3	348.9	572.9	420.0	50.1	31.6	58.2	46.6
Particles								
Number of particles evaluated	100.0	100.0	103.0	—	100.0	101.0	104.0	—
Mean number of particles (millions/lung lobes)	2.99	2.95	2.90	2.95	6.41	5.51	6.13	6.01^a^
≤1 μm particles (millions/lung lobes)	0.57	0.80	0.53	0.63	1.22	1.25	1.18	1.22
>1 μm to ≤3 μm particles (millions/lung lobes)	2.34	1.97	2.14	2.15	4.74	4.04	4.60	4.46
> 3μm particles (millions/lung lobes)	0.09	0.18	0.22	0.16	0.45	0.22	0.35	0.34

a The mean particle lung burden in group 3 does not include the large number of CaCO_3_ (limestone) particles that the rats in group 3 were exposed to, which as described earlier could not be counted as they were dissolved by the analytical procedure.

b WHO fibers are defined as having a length greater than 5.0 μm, a diameter less than 3.0μ m, and an aspect ratio equal to or greater than 3:1 (WHO, 1985).

**FIG. 7:**
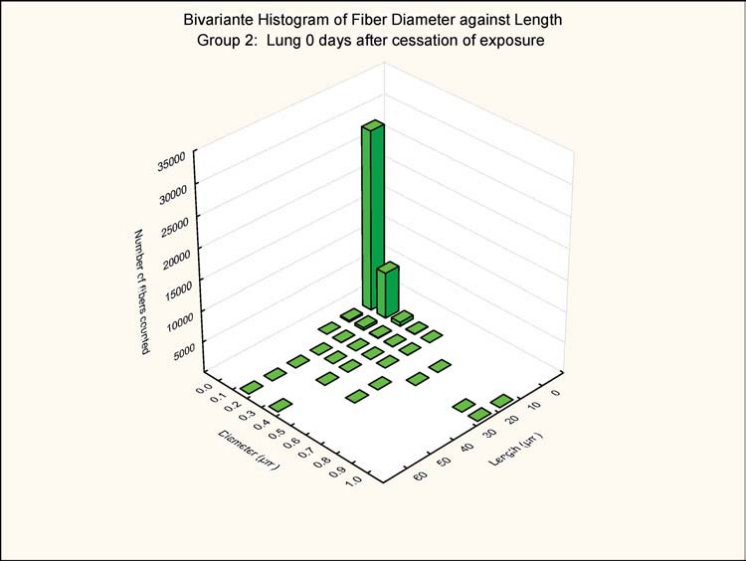
Group 2, chrysotile, bivariate length and diameter distribution measured of fibers recovered from the rat's lungs immediately after cessation of the 5-day exposure.

**FIG. 8:**
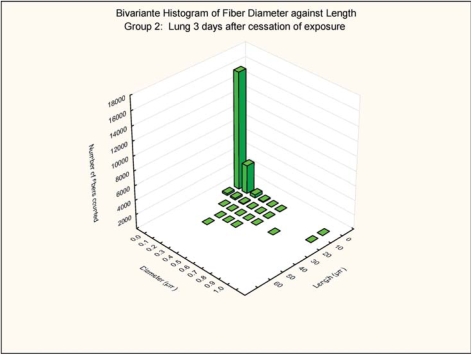
Group 2, chrysotile bivariate length and diameter distribution measured of fibers recovered from the rat's lungs 3 days after cessation of the 5-day exposure.

**FIG. 9:**
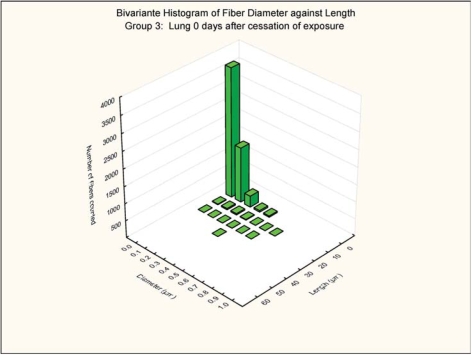
Group 3, chrysotile and sanded material, bivariate length and diameter distribution measured of fibers recovered from the rat's lungs immediately after cessation of the 5-day exposure.

**FIG. 10:**
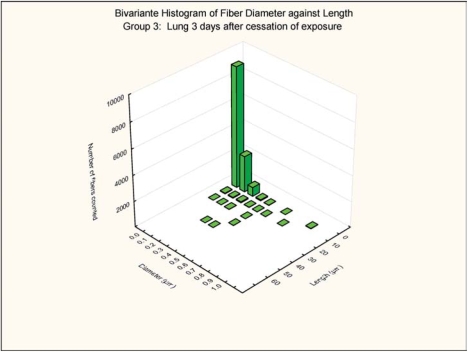
Group 3, chrysotile and sanded material, bivariate length and diameter distribution measured of fibers recovered from the rat's lungs 3 days after cessation of the 5-day exposure.

The bivariate length and diameter size distributions of the fibers remaining in the lungs at 0 and 3 days postexposure are shown in Figures 7, 8, 9, and 10.

### Fiber Clearance

**FIG. 11:**
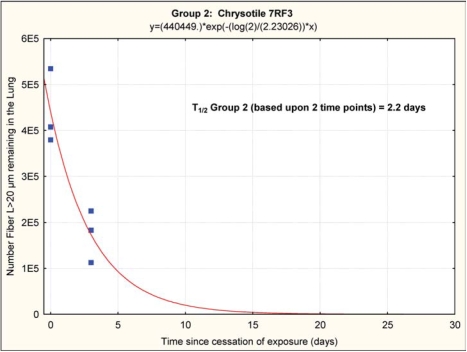
Group 2, chrysotile, clearance half-time of fibers longer than 20 μm of 2.2 days. It should be noted that these clearance half-times are based upon fitting an exponential clearance function to only two time points; however, they do provide a clear demonstration that the longer chrysotile fibers are rapidly disappearing from the lungs.

**FIG. 12:**
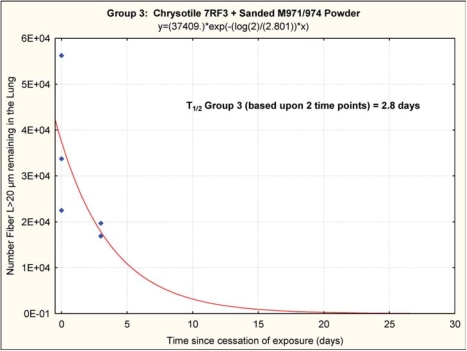
Group 3, chrysotile and sanded component, clearance half-time of fibers longer than 20 μm of 2.8 days. It should be noted that these clearance half-times are based upon fitting an exponential clearance function to only two time points; however, they do provide a clear demonstration that the longer chrysotile fibers are rapidly disappearing from the lungs.

The chrysotile fibers longer than 20 μm that deposited in the lungs rapidly “disappeared” from the lung, as shown in Figures 11 and 12, with a clearance half-time of fibers longer than 20 μm of 2.2 days (group 2, chrysotile) and 2.8 days (group 3, chrysotile and sanded component). It should be noted that these clearance half times are based upon fitting an exponential clearance function to only two time points; however, they do provide a clear demonstration that the longer chrysotile fibers are rapidly disappearing from the lungs. With only two time points and the interanimal variations in lung fiber burden, these two clearance half-times are not statistically different.

The clearance half-time of fibers 5-20 μm in length was 4.9 days for group 2 and 10.7 days for group 3. While these halftimes are not statistically different, it should be noted that the lung digestion technique includes all fibers in the lung and does not differentiate those fibers that would have been cleared to the lymphatics or bronchial airways.

### Histopathology

At necropsy, there were no findings that were considered to distinguish exposed rats from controls.

#### Microscopic Findings

**FIG. 13:**
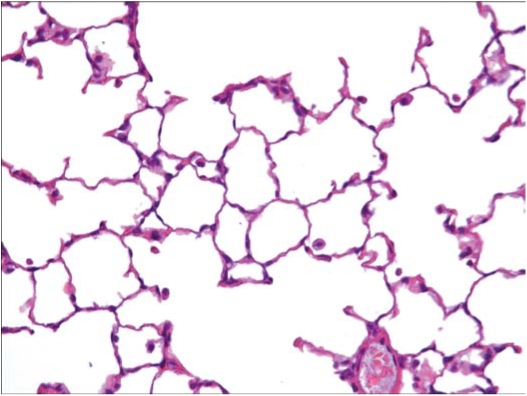
Histopathological photo of control lung from a group 1 rat exposed to filtered air using a similar generator and exposure system (40 × magnification). A few macrophages are seen.

**FIG. 14:**
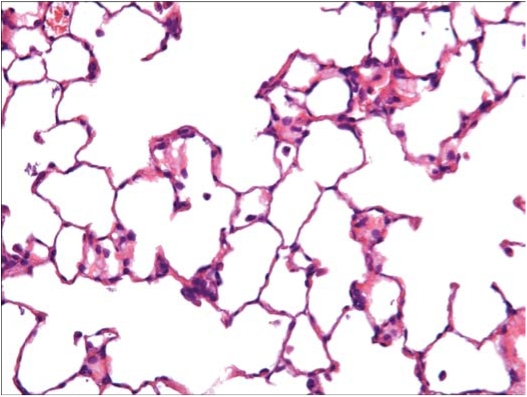
Histopathological photo of lung from a group 2 rat exposed to chrysotile alone (40 × magnification). A few macrophages are seen.

In the control rats exposed to filtered air (group 1), no findings considered to be exposure-related were noted. Figure 13 shows a histopathological photo of a group 1 rat. At sacrifice following day 5 of exposure, there were no findings considered to be exposure related in the rats exposed to chrysotile 7RF3 (group 2). Figure 14 shows a histopathological photo of a group 2 rat.

**FIG. 15:**
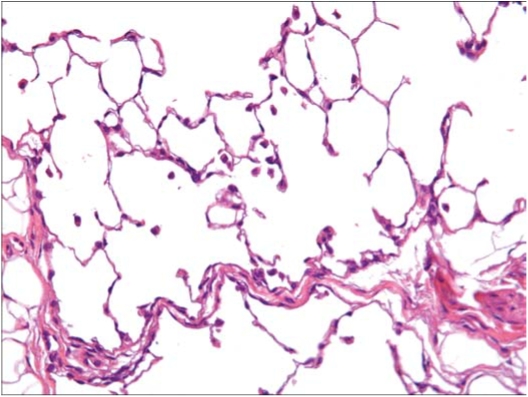
Histopathological photo of lung from a group 3 rat exposed to chrysotile and the sanded component (40 × magnification). An increased number of macrophages are observed with sometimes two or three per alveolus.

In the rats exposed to chrysotile 7RF3 mixed with sanded M971/974 powder, minimal alveolar macrophages were noted in 2 of the 3 rats, and mucus in bronchus, graded slight, was noted in 1 rat. While no fibers or particles were observed in either the macrophages or the mucus, this is most likely a result of the fine fiber diameters and the refractive index of the mounting media being close to that of the fibers. Figure 15 shows a histopathological photo of a group 3 rat.

The pathologist reported that the alveolar macrophages may be considered as a reaction to the inhaled fiber-powder mixture. The finding of a bronchial mucus plug was not considered to be related to the exposure.

At sacrifice 3 days after cessation of exposure, no findings considered to be exposure related were noted. This was considered by the pathologist to represent complete lung clearance for both the fiber and the fiber-powder mixture at this observation point.

## Discussion

### Biopersistence of 7RF3 Chrysotile

The clearance half-times of 2.2 and 2.8 days for the fibers *L* > 20 μm observed in this feasibility study are comparable to those reported for other commercial chrysotile fibers. Commercial Brazilian chrysotilewas reported to have a clearance half-time of 1.3 days (Bernstein et al., 2004), Calidria chrysotile was shown to have a clearance half-time of 0.3 days (Bernstein et al., 2005a), and Canadian chrysotile that was used for textile manufacturing had a clearance half-time of 11.4 days (Bernstein et al., 2005b). In these studies, the chrysotile alone was studied without the addition of any other components.

In the current comparative study, identical fiber aerosol generation systems were used for each exposure group with the only difference being that in group 3, a separate aerosol generator produced an aerosol of micronized sanded material that was added in the airstream to the chrysotile aerosol. The aerosol fiber number and fiber size distributions were similar in group 2 and 3 as presented earlier.

Care was taken in the experimental design to ensure that the fiber number and size distribution of the chrysotile in the aerosols of the two exposure groups were similar. Therefore, based upon the exposure aerosols, and the dynamics of lung deposition, the number and size of fibers deposited in the lungs was expected to be similar in both groups. However, the numbers of fibers remaining in the lungs at both the first and second sacrifice time points, immediately after the end of exposure and 3 days later, were quite different between the two exposure groups.

### Influence of the Sanded Aerosol on Fiber Clearance

As has been described previously, chrysotile fiber clearance is related to the interaction of the mineral structure of the fiber with the lung environment and cells (Bernstein & Hoskins, 2006). Long fibers with low biosolubility are the most pathologically active fiber size category, and clearance of these fibers is very slow due to the inability of macrophages to phagocy-tize them completely, leading to frustrated phagocytosis (Oberdorster, 2000). Chrysotile is a rolled silicate sheet with magnesium on the outside of the sheet and silica on the inside. Longer chrysotile fibers are shortened in the lungs and this is considered to be a consequence of the action of the lung environment on the chemical structure of the chrysotile fibers. The dual action of both the lung surfactant and the acid environment of the macrophage phagolysosome may leach the magnesium layer and break apart the silica bonds. This causes longer fibers to break into smaller fibers and particles, which can then be fully phagocytized and cleared by the macrophage.

One of the earlier descriptions of the colloidal and surface chemistry of chrysotile was presented by Pundsack (1955) based upon differential thermal analysis and thermo-gravimetric dehydration characteristics of chrysotile. He described how similarities of certain aspects of the chemical behavior of chrysotile to that of magnesium hydroxide are shown to be compatible with the crystal structure of the mineral. Pundsack reported how in contact with relatively pure water the fiber surface dissociates partially until an equilibrium on the order of that attained by pure magnesium hydroxide is reached. He also described how chrysotile reacts with strong acids to form a hydrated silica residue, which resulted in an important transition that he described as increased turbidity when the pH approaches 4.

Thomassin et al. (1977) investigated the dissolution kinetics of chrysotile by studying with electron spectrometry (XPS = ESCA), the variations in the surface composition of chrysotile fibers that have been leached for various lengths of time in a nonstirred and non-renewed medium. He described two models at acid pH (0.1 N Oxalic Acid) and a range of temperatures (22-80°C) that could explain the dissolution of chrysotile: diffusion of Mg^2+^ through the fibrous gel, or dissociation of chrysotile. If the dissociation of chrysotile is considered the rate-limiting step, Thomassin determined the relationship between leached thickness rate and temperature to be exponential [*V*(Å/h) = 0.5629 × exp(0.0767 × Temp)]. At 37°C the leached thickness increases at 9.6 Å/h. For example, if the chrysotile fiber is 0.2 μm thick (= 2000 Å) and assuming the leaching is symmetrical (occurs from both sides of the fiber), the fiber would fully dissociate in 104 h or 4.3 days. Thomassin et al. (1977) also report that an acid attack of chrysotile produces ”cracks” or ”channels,” as indicated by the continuous increase of the specific area with leaching. Such formations could further facilitate the breaking apart of longer chrysotile fibers.

### Macrophages and Biopersistence

The finding that the total number of fibers present in the lungs of the animals in group 3 (chrysotile and sanded material) is approximately an order of magnitude less than in group 2 (chrysotile alone) is intriguing. This difference was evident immediately at the end of the 5-day exposure period, suggesting that there had been accelerated clearance during ongoing exposure to the combined fiber/particle exposure. This finding is the opposite of that seen with 'rat lung overload,' where concomitant exposure to particulate at high exposure concentrations has been reported to prolong clearance half-time, with pathological consequences (Bolton et al., 1983; Muhle et al., 1988; Morrow, 1988,1992; Oberdorster, 1995). However, the present exposure would have produced a much lower lung burden than is associated with rat lung overload (Oberdorster, 1995), where overload commonly follows weeks of exposure to an airborne mass concentration 10 or more times the level used here.

In seeking an explanation for this rather paradoxical finding of increased clearance of fibers during concomitant exposure to particles in group 3, we noted that histopathological examination of the lungs showed increased macrophage numbers in group 3 as compared to group 2. The pathologist reported that the increased number of ”alveolar macrophages (in group 3) may be considered as a reaction to the inhaled fiber-powder mixture.” This macrophage inflammation would be expected in response to the greater number of fine particles that were deposited in the lungs of the animals in group 3. As shown in Table 5, immediately after cessation of the 5 days of exposure there was a mean lung burden of 7.7 million particles per lung in group 3, in comparison to 3.3 million particles in group 2. However, it should be noted that the mean particle lung burden in group 3 does not include the large number of CaCOs (limestone) particles that the rats in group 3 were exposed to, which, as described earlier, could not be counted as they were dissolved by the analytical procedure. It is well known that long biopersis-tent fibers are not efficiently cleared from the lungs (Hesterberg et al., 1994, 1998; Searl et al., 1999), while non-biopersistent fibers, like chrysotile (Bernstein & Hoskins, 2006), are cleared. The fact that there is greater clearance of the long chrysotile fibers in the combined exposure group means that there must be more rapid leaching and breakage of these long fibers as the macrophages attempt to engulf them. The accelerated disintegration of the chrysotile fibers in the lungs of the rats receiving the combined exposure could be explained by two factors: the increased numbers of macrophages present in these lungs, and the macrophages could have a greater ability to cause leaching and breakage. The greater numbers of macrophages mean that the total amount of long fiber inside macrophage phagosomes is increased, and so the total ”acid stress” applied to these chrysotile fibers is greater. The reasoning that there is greater potential for individual macrophages to cause disintegration is supported by the finding of a mild degree of inflammation, as indicated by the increased numbers of macrophages seen in the lungs of the combined group. Exposure to particles leads to the induction of inflammation (Silver et al., 1988; Schaible et al., 1998; Haas, 2007), and a macrophage infiltrate typical of inflammation was documented in the lungs of the combined exposure. During inflammation macrophages undergo ”activation,” a change in differentiation status to more active secretory and functional pheno-type. Of special relevance here is the finding that phagosomes of activated macrophages rapidly become more acidic than those of resting macrophages by about 1 pH unit (Schaible et al., 1998).

**FIG. 16:**
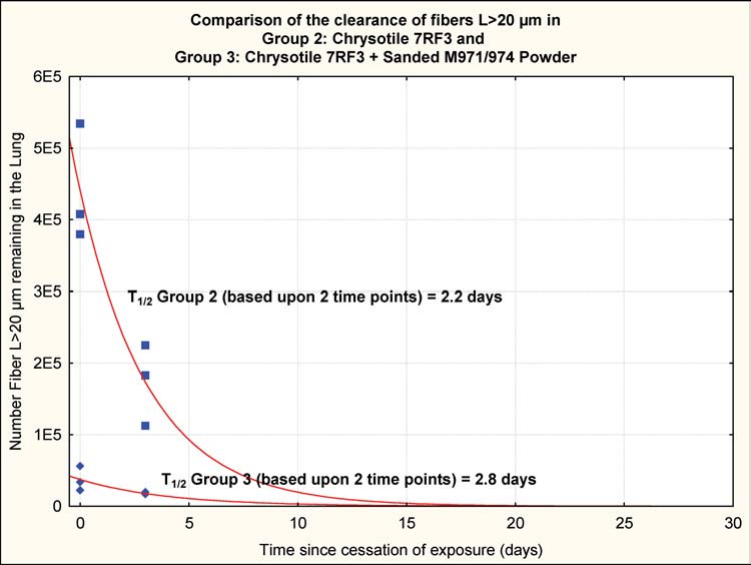
Clearance curve from group 2 (chrysotile) and group 3 (chrysotile and sanded material).

In addition, particles less than 0.4 μim in diameter are more likely to be taken up by the macrophage by endoyctosis rather than by phagocytosis (Hess, 2007; Champion & Mitragotri, 2006), while larger particles would be preferentially phagocy-tized. Thus, the larger particles of the sanded material (GMD = 1.18 μm) would augment the phagocytic response of the macrophage, a process that would stimulate further the acidic environment.

Since pH is a logarithmic scale, the activated macrophages in the combined exposure group could have the chrysotile fibers enclosed in phagosomes that are much more acidic than those in the chrysotile-alone group. The greater activation of macrophages in the combined exposure group, with a more acidic milieu being applied to the chrysotile fibers in phagosomes bringing about their accelerated disintegration, may therefore explain the decreased biopersistence of the chrysotile in that group.

This shift in biopersistence is illustrated in Figure 15, which shows the clearance curve from group 2 (chrysotile) and group 3 (chrysotile and sanded material).

## Conclusions

This feasibility study has shown that across all size ranges there was approximately an order of magnitude decrease in the mean number of each size category of fibers remaining in the lung in group 3 as compared to group 2 at the end of the exposure, even though the fiber aerosol exposures were closely similar. This can only be explained by more efficient clearance of the long fibers during the ongoing combined exposure in group 3 as compared to the chrysotile alone in group 2. Starting from these different postexposure lung fiber burdens, the chrysotile in the group 2 rats alone was rapidly removed from the lung with a clearance half-time 2.2 days for the fibers *L* > 20 μm, while the clearance halftime of fibers *L* > 20 μm in the group 3 rats was 2.8 days.

The histopathological examination showed that the lungs of the chrysotile-exposed animals were not different from those of the air controls, while there was a typical dust-related macrophagic inflammation in the airspaces of the group 3 animals. The greater numbers of macrophages and potentially a more acidic internal environment could explain the more rapid shortening and clearance of the chrysotile during ongoing exposure in group 3.

This study uniquely illustrates that with the addition of an aerosol of the sanded joint particulate, the larger number of fine particles deposited in the lung accelerates the recruitment of macrophages, resulting in a 10-fold decrease in the number of fibers across all size ranges remaining in the lung. The increased number of macrophages was confirmed histologically, with this being the only exposure-related finding reported.
